# Negative Capacitance Vacuum Channel Transistors for Low Operating Voltage

**DOI:** 10.3390/mi11060543

**Published:** 2020-05-27

**Authors:** Woo Young Choi

**Affiliations:** Department of Electronic Engineering, Sogang University, Seoul 04107, Korea; wchoi@sogang.ac.kr; Tel.: +82-2-715-8467

**Keywords:** negative capacitance, ferroelectric capacitor, capacitance matching, NC vacuum channel transistor, vacuum channel transistor, steep switching, hysteresis effects

## Abstract

This study proposes negative capacitance vacuum channel transistors. The proposed negative capacitance vacuum channel transistors in which a ferroelectric capacitor is connected in series to the gate of the vacuum channel transistors have the following two advantages: first, adding a ferroelectric capacitor in series with a gate capacitor makes the turn-on voltage lower and on–off transition steeper without causing hysteresis effects. Second, the capacitance matching between a ferroelectric capacitor and a vacuum channel transistor becomes simplified because the capacitance of a vacuum channel transistor as seen from a ferroelectric capacitor is constant.

## 1. Introduction

Over the past 60 years of the semiconductor industry, the size of metal-oxide-semiconductor field-effect transistors (MOSFETs) has been scaled down obeying Moore’s law: feature sizes of transistors are scaled at a rate of approximately 0.7 times every 18 months. As the semiconductor market size increases, its applications extend beyond consumer electronics, extending, for example, to transistors, microchips, solar cells, and light-emitting diodes. Recently, electronic devices have faced burgeoning demand from aerospace and extreme-environment applications. For example, in the case of aerospace applications, many kinds of challenges from harsh environments exist. Extremely low and high temperature and high levels of cosmic ray and radiation lead to catastrophic damage to the electronic systems without proper shielding packages. Unfortunately, it is well-known that MOSFETs, which are the most widely used electronic devices, are difficult to use for these applications because they are vulnerable to radiation and temperature [1,2,3,4,5,6,7,8,9,10,11]. Even if state-of-the-art shielding methods can protect MOSFETs from harsh environments, the following issues still remain: large volume, large weight, high power consumption, and complex system design.

Thus, as an alternative, a vacuum channel transistor has been proposed, which replaces the semiconductor channel with a vacuum channel. Owing to the unique properties of a vacuum channel, vacuum channel transistors are robust even if they are exposed to radiation and high or low temperatures [12]. Furthermore, it is well-known that a vacuum environment is superior to a solid one in terms of carrier transport because ballistic transport is feasible in the former while the latter experiences various scattering mechanisms, such as lattice vibration scattering, ionized impurity scattering, surface roughness scattering, etc. [13]. For example, the electron velocities in vacuum and silicon are theoretically 3 × 10^10^ cm/s and ~10^7^ cm/s. Thus, vacuum channel transistors have the potential to implement higher performance and power gain than MOSFETs. In spite of the above-mentioned advantages, vacuum channel transistors experience a high operating voltage (*V*_DD_) [14], which is due to their current flowing mechanism: Fowler–Nordheim tunneling, where electrons tunnel through the barrier in the presence of a high electric field [15,16]. Because Fowler–Nordheim tunneling makes electrons tunnel through a rounded triangular barrier generated at the source-to-channel junction, electrons tunnel from the source tip into the vacuum channel with a positive drain voltage (*V*_D_) when the gate voltage (*V*_G_) exceeds the turn-on voltage. In the case of the optimized vacuum channel transistors, an insulated-gate structure with a pyramidal source and a flat drain is introduced to boost the on-current (*I*_on_) and gate controllability by increasing the local electric field [17,18]. However, despite the optimization and downscaling of vacuum channel transistors, their *V*_DD_ is still higher than that of MOSFETs, which makes them hard to utilize in low-power extreme-environment applications.

**Figure 1:**
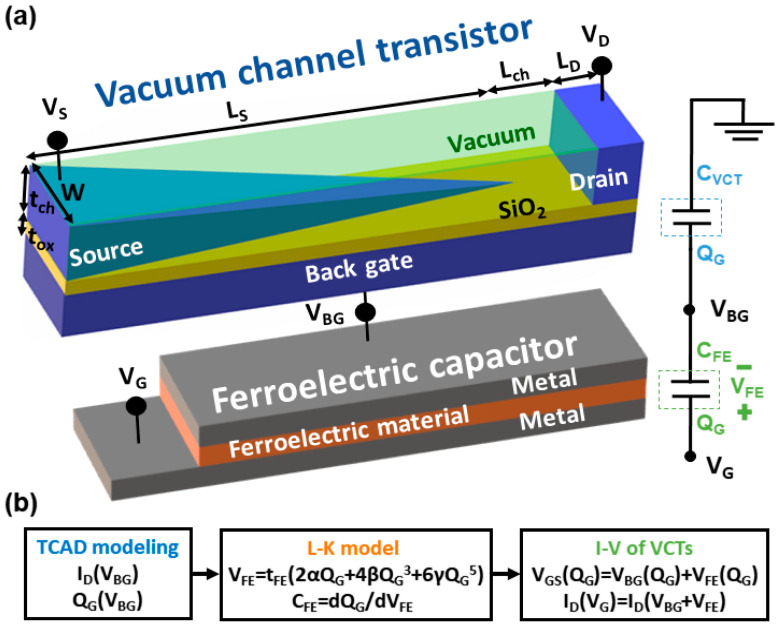
(**a**) Bird’s eye view of the proposed negative capacitance vacuum channel transistor and its equivalent capacitor network model. The channel length (*L*_ch_), source length (*L*_S_), drain length (*L*_D_), and width (*W*) are 10 nm, 200 nm, 10 nm, and 30 nm, respectively. (**b**) Simulation procedure of negative capacitance vacuum channel transistors combining technology computer-aided design (TCAD) simulation results with the Landau–Khalatnikov (LK) equation.

In this paper, for a low *V*_DD_, a negative capacitance vacuum channel transistor is proposed for the first time, as shown in Figure 1a. The gate of a vacuum channel transistor is connected to a ferroelectric capacitor to combine the advantages of vacuum channel transistors with negative capacitance. The negative capacitance effects of ferroelectric materials have recently been exploited to induce internal voltage gain out of the gate stack [19]. The underlying physics for an abrupt on–off switching operation of a negative capacitance transistor is the passive amplification of the gate voltage at the interface between the FE gate oxide and the semiconductor channel. Essentially, the charge balance between the series-connected ferroelectric and linear positive capacitor induces a depolarization field and stabilizes the ferroelectric capacitor at a negative capacitance state that in turn amplifies the surface potential of the electron device. It has been reported that there are two important aspects of a negative capacitance transistor: amplification and stabilization. The differential amplification of the gate voltage at the interface between the surface channel and the gate oxide makes the on–off transition of a negative capacitance transistor more abrupt. On the other hand, negative capacitance is unstable by nature. Thus, the positive capacitances can stabilize the ferroelectric in its negative capacitance state, leading to a stable voltage amplification. Conventionally, a metallic layer is located between the gate and vacuum channel transistor to average out the nonuniform potential profile along the source-drain direction and the charge nonuniformity coming from domain formation in the ferroelectric. It makes the single-domain Landau–Khalatnikov (LK) equation valid. The influence of the internal voltage gain stemming from negative capacitance has also been recently confirmed in the case of polymer ferroelectric bulk MOSFETs [20,21,22,23], negative capacitance finFETs [24,25,26], and negative capacitance nanoelectromechanical (NEM) relays [27]. This manuscript is the first application of negative capacitance to vacuum channel transistors whose channel is vacuum rather than semiconductor.

The proposed negative capacitance vacuum channel transistors have the following two benefits: first, adding a ferroelectric capacitor in series with a gate dielectric capacitor makes the turn-on voltage lower and on–off transition more abrupt without causing unwanted hysteresis effects. Second, the capacitance matching between a ferroelectric capacitor and a vacuum channel transistor is simplified because the capacitance of a vacuum channel transistor as seen from a ferroelectric capacitor is constant. When a vacuum channel transistor is connected to ferroelectric materials, the capacitance matching between the ferroelectric-layer (*C*_FE_) and the vacuum channel transistor (*C*_VCT_) is important. As capacitance matching is improved, the subthreshold swing (*SS*) and transconductance of vacuum channel transistors improve, which leads to a lower *V*_DD_ [28]. For the optimized matching condition, *C*_VCT_^−1^ + *C*_FE_*^−^*^1^ needs to be made as small as possible while maintaining positive values for all charges to minimize *SS* and avoiding hysteresis effects: *C*_VCT_ (*Q*_G_)^−1^ ≥ −*C*_FE_ (*Q*_G_)^−1^ [20], where *Q*_G_ is the gate charge. However, in the case of MOSFETs, their gate capacitance is dependent on bias conditions, which makes the capacitance matching of negative capacitance MOSFETs difficult [29]. For example, as *V*_G_ increases, the gate capacitance of n-channel MOSFETs increases nonlinearly from the subthreshold to a strong inversion. In contrast, the capacitance matching of negative capacitance vacuum channel transistors is expected to be simplified and stable because there is no semiconductor channel region. The ferroelectric capacitor in standalone condition cannot show the negative capacitance behavior because to stabilize the total system, it is necessary to introduce a series combination of a ferroelectric capacitor and a linear positive capacitor connected to a voltage source. The *C*_VCT_ remains constant regardless of the *Q*_G_, because the *Q*_G_ of the vacuum channel transistors increases linearly with the increment of the back-gate voltage (*V*_BG_). The advantages of negative capacitance vacuum channel transistors are discussed in detail in the following section.

## 2. Simulation Method

Figure 1b summarizes the simulation procedure of the negative capacitance vacuum channel transistors. First, the drain current (*I*_D_) and *Q*_G_ of vacuum channel transistors are calculated as a function of *V*_BG_ using a commercial three-dimensional technology computer-aided design (TCAD) simulator [30]. The simulation models include band-to-band tunneling, Fowler–Nordheim tunneling, Fermi distribution, Shockley-Read-Hall (SRH) recombination, and dynamic nonlocal tunneling models. HfSiO and SiO_2_ are used as ferroelectric and gate dielectric materials, respectively. The work function of the back-gate, source, and drain is 4.32 eV, which corresponds to that of tungsten. The physical parameters of the simulated negative capacitance vacuum channel transistors are as follows: the channel (*t*_ch_) and oxide thickness (*t*_ox_) are 15 nm and 5 nm, respectively. The source length (*L*_S_), channel length (*L*_ch_), and channel width (*W*_ch_) are 200 nm, 10 nm, and 30 nm, respectively. Parasitic capacitance components of vacuum channel transistors are included in the TCAD simulation, while the leakage through the ferroelectric layer is ignored for concise discussion. Second, to derive the voltage drop (*V*_FE_) and capacitance (*C*_FE_) across the ferroelectric capacitor, the LK equation is coupled with TCAD simulation using Equations (1) and (2) [20].

(1) $$V_{\mathrm{FE}}=t_{\mathrm{FE}}\left(\alpha_{0}Q_{G}+\beta_{0}Q_{G}^{3}+\gamma_{0}Q_{G}^{5}\right)$$

(2) $$C_{\mathrm{FE}}=dQ_{G}/dV_{\mathrm{FE}}=1/t_{\mathrm{FE}}\left(\alpha_{0}Q_{G}+3\beta_{0}Q_{G}^{2}+5\gamma_{0}Q_{G}^{4}\right)$$

where *α*_0_, *β*_0_, and *γ*_0_ refer to the Landau coefficients of HfSiO (*α*_0_ = −1.73 × 10^9^ m/F, *β*_0_ = 7.68 × 10^10^ m^5^/F/C^2^, and *γ*_0_ = 0 m^9^/F/C^4^, as presented in [31]). The value of *α*_0_ is negative for all known ferroelectric materials below Curie temperature, which leads to hysteretic characteristics of ferroelectrics. It shows the double-well shape of free energy for negative capacitance behavior of ferroelectric capacitors. *t*_FE_ is the ferroelectric layer thickness. Equations (1) and (2) calculate *V*_FE_ and *C*_FE_ by using *Q*_G_ values obtained in the first step. Finally, the calculated *V*_FE_ is added to *V*_BG_ to obtain *V*_G_, as shown in Figure 1a. Then, simulated *I*_D_ vs. *V*_G_ and *I*_D_ vs. *V*_D_ curves of negative capacitance vacuum channel transistors are generated.

## 3. Simulation Results

**Figure 2:**
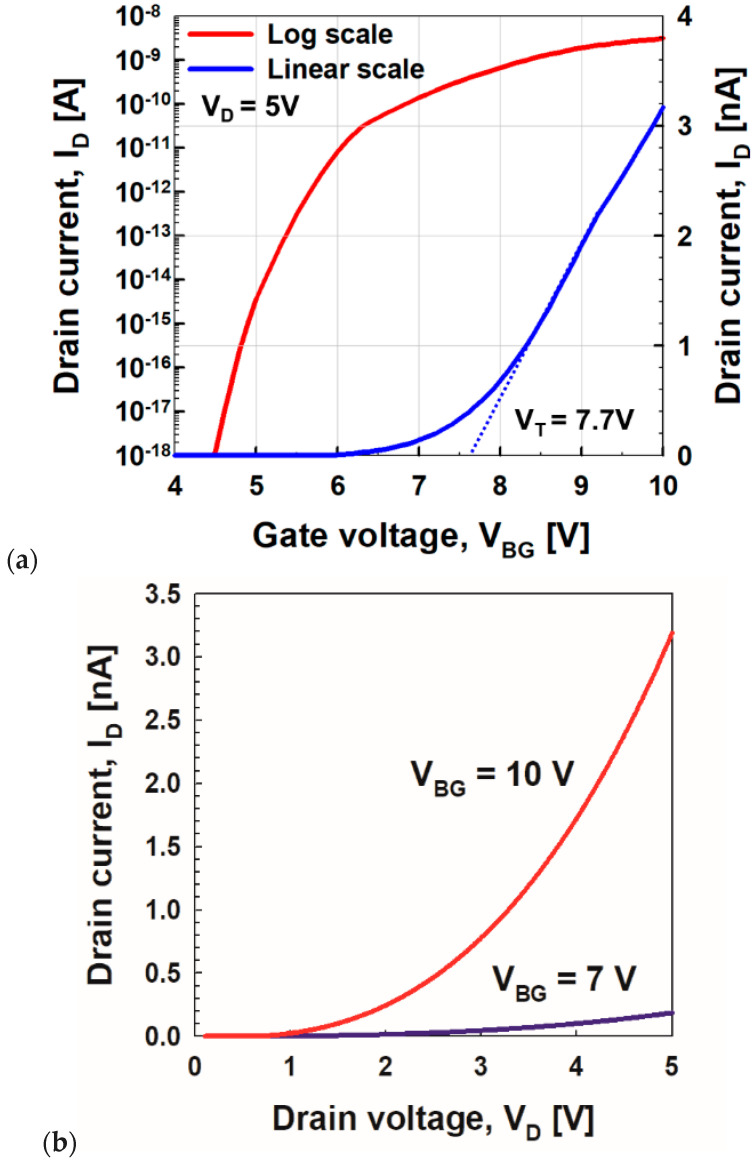
Simulated (**a**) drain current (*I*_D_) vs. back-gate voltage (*V*_BG_) and (**b**) *I*_D_ vs. drain voltage (*V*_D_) without ferroelectric capacitors.

**Figure 3:**
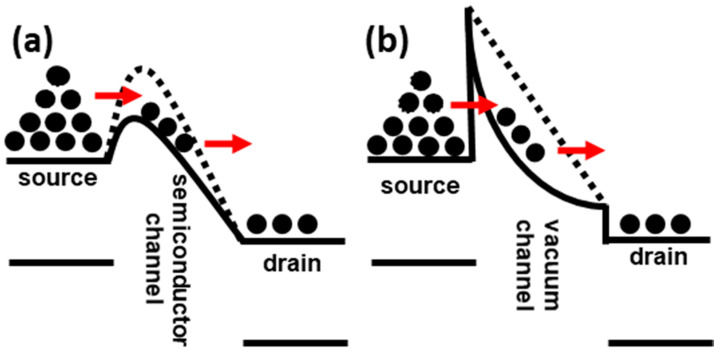
Energy band diagrams of a (**a**) metal-oxide-semiconductor field-effect transistors (MOSFET) and (**b**) vacuum channel transistor. The carrier transport mechanism of a MOSFET is thermionic emission, while that of a vacuum channel transistor is Fowler–Nordheim tunneling, respectively.

Figure 2 shows the *I*_D_ vs. *V*_BG_ and *I*_D_ vs. *V*_D_ curves of a vacuum channel transistor, which corresponds to negative capacitance vacuum channel transistors without ferroelectric capacitors. The output curves in Figure 2b show the typical current vs. voltage characteristics of Fowler–Nordheim tunneling. Thus, *V*_BG_ rather than *V*_G_ controls the vacuum channel transistor. As previously reported, the dominant carrier transport mechanisms of vacuum channel transistors are Fowler–Nordheim tunneling and thermionic emission [13], as shown in Figure 3. In the case of vacuum channel transistors, the semiconductor channel is replaced with a vacuum channel. In the case of MOSFETs, carriers stored in the source region are injected into the semiconductor channel region by lowering the energy barrier height using the *V*_G_. Then, the carriers move from the source into the semiconductor channel using thermionic emission: high-energy carriers following the Fermi–Dirac distribution are injected over the energy barrier. Subsequently, the carriers move along the channel while experiencing scattering events, which are described by a carrier mobility. On the contrary, in the case of vacuum channel transistors, the carriers stored in the source need to overcome the energy barrier between the source and vacuum channel. Fowler–Nordheim tunneling is a more viable option than thermionic emission, because the vacuum level is significantly higher than the energy level of the semiconductor. Once the *V*_BG_ is high enough to narrow the source-to-channel barrier width, the source carriers begin to be injected into the channel region. After the injection, the carriers drift through the channel into the drain without experiencing scattering events analogous to ballistic transport in extremely short-channel semiconductor MOSFETs. During this process, vacuum channel transistors generally experience a high *V*_DD_. For efficient source carrier injection, the electric field or energy band bending near the source-to-channel junction must be increased. To meet this condition, the source tip is sharpened, as shown in Figure 1a, and a multiple-gate structure is introduced. However, these geometrical approaches are insufficient to obtain a dramatic reduction in *V*_DD_ and *SS*. Thus, the introduction of a ferroelectric capacitor is helpful for alleviating the weal spots of vacuum channel transistors.

**Figure 4:**
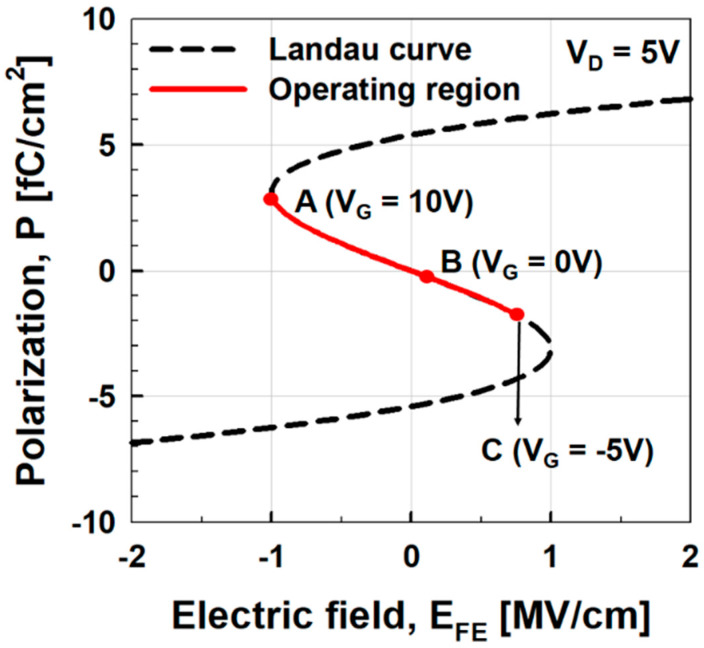
Polarization (*P*) vs. electric field (*E*_FE_) of the ferroelectric material, HfSiO. Solid line shows the operating regions of negative capacitance vacuum channel transistors. Points A, B, and C represent position on Landau curve for *V*_G_ = 10 V, 0 V, and −5 V, while *V*_D_ is fixed at 5 V.

In Figure 2, the threshold voltage (*V*_T_) extracted by the linear extrapolation method is ~7.7 V at *V*_D_ = 5 V. Even if *V*_G_ increases up to 10 V, *I*_D_ only reaches ~3 nA per source tip. Then, ferroelectric materials such as HfSiO are connected in series with the back-gate stack of negative capacitance vacuum channel transistors whose polarization (*P*) vs. electric field across a ferroelectric layer (*E*_FE_) curve is shown in Figure 4. On the Landau curve, the operating points corresponding to *V*_D_ = 5 V and *V*_G_ = −5–10 V are shown. If the operating point is located at A, it lowers the gate voltage (*V*_G_ = *V*_BG_ + *V*_FE_) required to reach the same value of *I*_D_. This leads to a higher *I*_on_ and lower turn-on voltage. At operating point B, *E_FE_* is still positive, which means that *V*_BG_ is negative even when *V*_G_ = 0 V. This leads to a lower off-current (*I*_off_).

**Figure 5:**
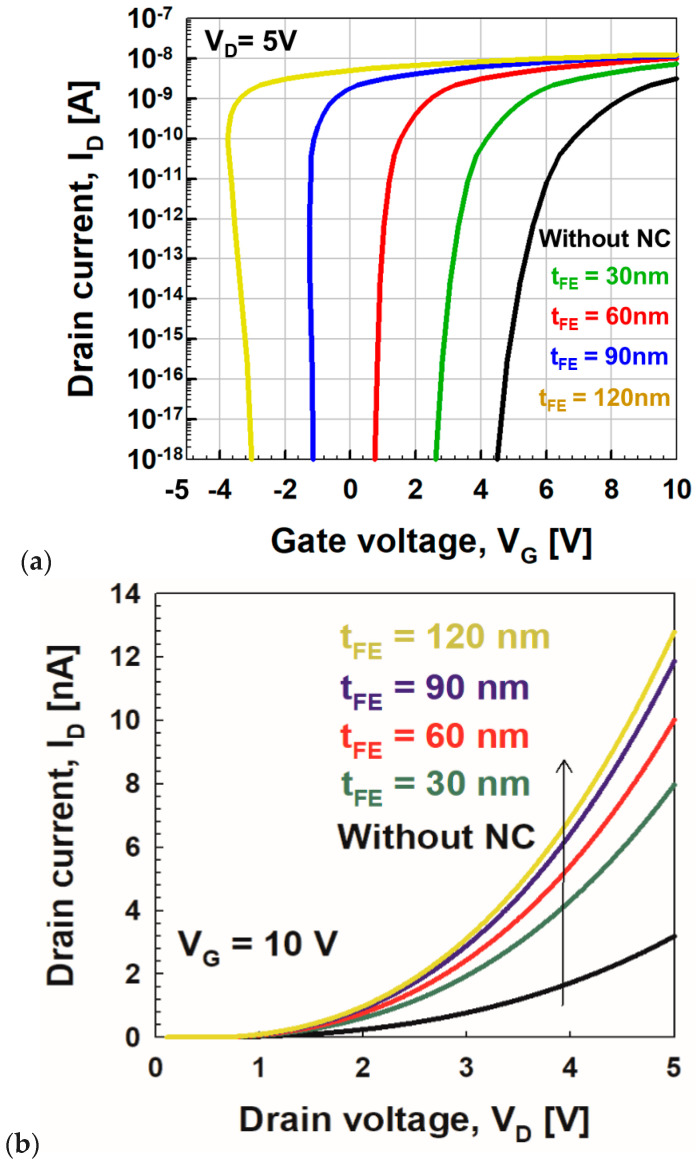
Simulated (**a**) *I*_D_ vs. *V*_G_ and (**b**) *I*_D_ vs. *V*_D_ curves of negative capacitance vacuum channel transistors for various ferroelectric thicknesses (*t*_FE_ = 30 nm, 60 nm, 90 nm, and 120 nm). The S-shape of thicker than 60-nm-thick ferroelectric-HfSiO indicates the hysteresis behavior.

Figure 5 shows the influence of the ferroelectric capacitor on the transfer and output curves of negative capacitance vacuum channel transistors with the variation of *t*_FE_. This clearly shows the performance boosting of negative capacitance vacuum channel transistors as *t*_FE_ increases. The surface potential of vacuum channel transistors can be higher than the applied *V*_G_, resulting in negative capacitance effects of the ferroelectric material. As *t*_FE_ increases, turn-on voltage decreases, *I*_on_ increases, and *SS* improves. Among the values of *t*_FE_, 60 nm is considered to be an optimal value because minimal *SS* is achieved without hysteresis effects, whereas turn-on voltage is <1 V. If *t*_FE_ exceeds 60 nm, the hysteresis operation featuring S-shaped transfer curves becomes more pronounced.

## 4. Discussion

**Figure 6:**
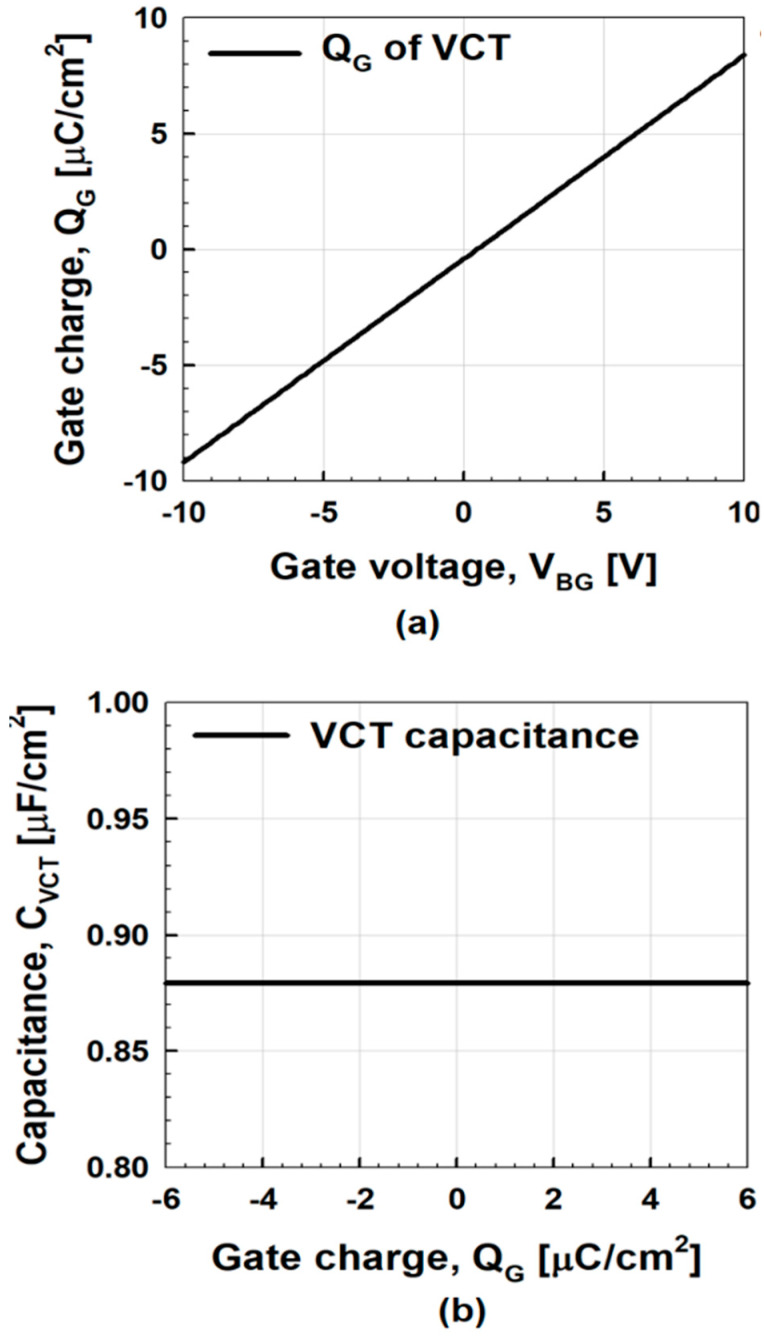
(**a**) *V*_BG_ vs. *Q*_G_ and (**b**) vacuum channel transistor (*C*_VCT_) vs. *Q*_G_ of vacuum channel transistors.

**Figure 7:**
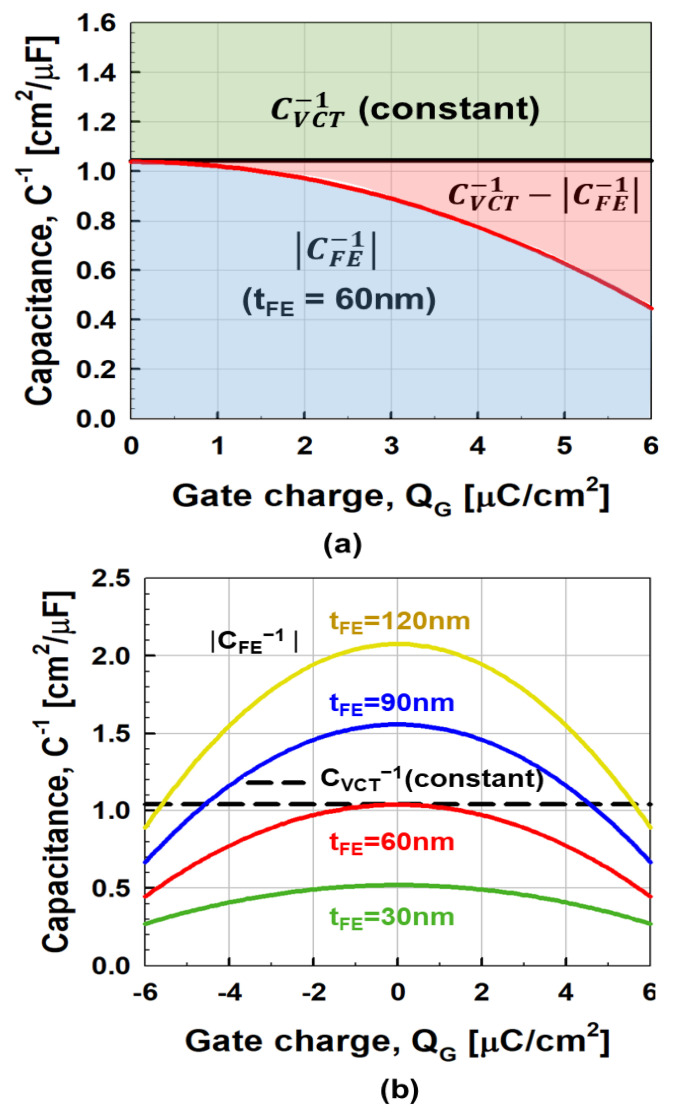
Comparison of vacuum channel transistor capacitance vs. ferroelectric capacitance as function of *Q*_G_ for (**a**) ferroelectric thickness (*t*_FE_) is 60 nm, and (**b**) various ferroelectric thicknesses (*t*_FE_ = 30 nm, 60 nm, 90 nm, and 120 nm). The calculated capacitances are according LK model.

**Figure 8:**
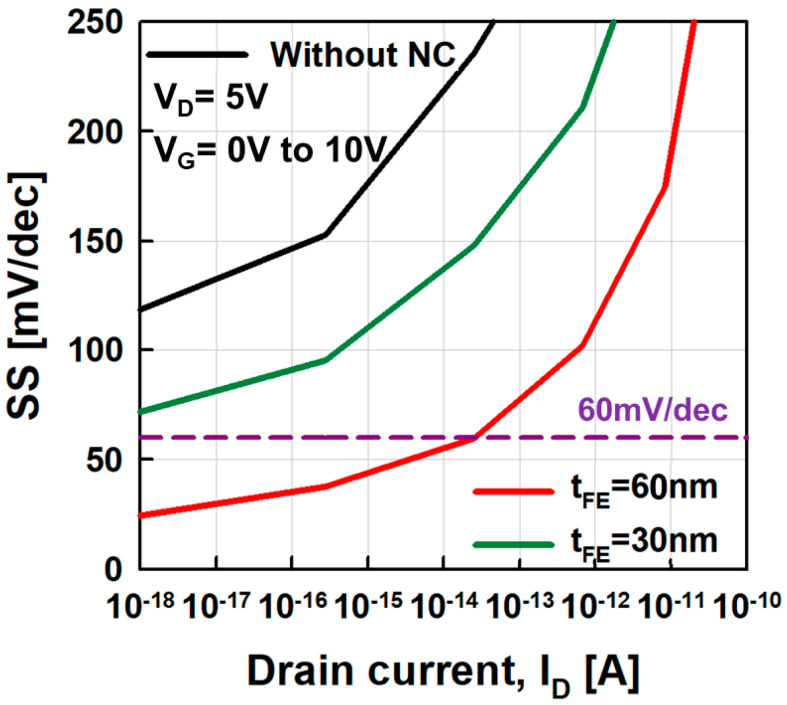
*SS* as a function of *V*_G_ for different *t*_FE_ (0 nm, 30 nm, and 60 nm).

For more detailed analysis, Figure 6a,b shows *Q*_G_ vs. *V*_BG_ and *C*_VCT_ vs. *Q*_G_ of vacuum channel transistors. It should be noted that *C*_VCT_ remains constant regardless of bias conditions unlike negative capacitance MOSFETs. Although *C*_FE_ is a nonlinear function of *Q*_G_, it can be matched with *C*_VCT_ around zero *Q*_G_. It means that perfect capacitance matching between *C*_VCT_ and *C*_FE_ is easier in the case of negative capacitance vacuum channel transistors than negative capacitance MOSFETs: minimizing *SS* without causing hysteresis effects as shown in Figure 7a,b. Considering the equivalent capacitance model in Figure 1a, the body factor (*m*) of negative capacitance vacuum channel transistors can be expressed as [19]

(3) $$m=1+\frac{C_{\mathrm{VCT}}}{C_{\mathrm{FE}}}=\frac{C_{\mathrm{VCT}}^{-1}-\left(-C_{\mathrm{FE}}^{-1}\right)}{C_{\mathrm{VCT}}^{-1}}$$

which determines the coupling between the *C*_VCT_ and *C*_FE_. For the minimization of *m* without hysteresis effects, the following two requirements must be met: first, total capacitance (*C*_total_) should remain positive in the entire range of operation; *C*_total_^−1^ = *C*_FE_^−1^ + *C*_VCT_^−1^ ≥ 0, which means *C*_VCT_^−1^ ≥ −*C*_FE_^−1^ [22]. Second, *C*_total_^−1^ should be made as small as possible [20]. As shown in Figure 7a, as *C*_VCT_^−1^, which is independent of *Q*_G_, decreases down to *C*_FE_^−1^, the gap between *C*_VCT_^−1^ and *C*_FE_^−1^ becomes narrower, which leads to a reduced *SS*. For example, in Figure 7a, the value of *C*_VCT_^−1^ is constant: 1.04 cm^2^/μF. In contrast, when *V*_G_ is 0.4 V and *t*_FE_ is 60 nm, the value of −*C*_FE_^−1^ is 1.03 cm^2^/μF. This implies that *m* = 0 is feasible by adjusting *t*_FE_. Thus, Figure 5 shows that the increase in *I*_D_ is steepest near *V*_G_ = 0.4 V when *t*_FE_ is optimized to 60 nm. Figure 7b shows the relationship between *C*_VCT_^−1^ $\left|C_{\mathrm{FE}}^{-1}\right|$ and *Q*_G_ with the variation of *t*_FE_. As *t*_FE_ becomes greater than 60 nm, −*C*_FE_^−1^ exceeds *C*_VCT_^−1^ near *Q*_G_ = 0, making two intersections, which lead to hysteresis effects or the S shape of the transfer curves. On the contrary, if *t*_FE_ becomes less than 60 nm, and −*C*_FE_^−1^ becomes less than *C*_VCT_^−1^ for all *QGs*. Even if no hysteresis effect is observed, *SS* reduction is limited. Figure 8 shows the *SS* vs. *I*_D_ curves under the three *t*_FE_ conditions. As *t*_FE_ increases, *SS* decreases. If *I*_D_ is fixed at 10^−9^ nA, *SS* becomes 118 mV/dec, 74 mV/dec, and 25 mV/dec at *t*_FE_ = 0 nm, 30 nm, and 60 nm, respectively. As shown in Figure 5, the 60-nm-*t*_FE_ case shows minimal *SS* without causing hysteresis effects.

## 5. Conclusions

In this paper, the low-voltage operation of negative capacitance vacuum channel transistors are simulated using the unique property of ferroelectric materials. In addition, a negative capacitance effect is achieved for abrupt on–off transition without causing hysteresis effects through capacitance matching to stabilize the total system. The ferroelectric capacitor can amplify the *V*_BG_, the *SS* can be lowered, and the *I*_D_ vs. *V*_G_ curve steepens. The operation voltage can be lowered below 1 V at the 60-nm thickness HfSiO without showing hysteresis behavior. The *Q*_G_ of the vacuum channel transistor is constantly increased as *V*_BG_ increases, and *C*_VCT_ is constant as *Q*_G_ increases. Thus, the negative capacitance vacuum channel transistor is relatively simple to match the vacuum channel transistor capacitance and ferroelectric capacitance. Therefore, the *SS* characteristic is better because the difference between *C*_VCT_^−1^ and −*C*_FE_^−1^ is reduced compared to the solid-state device with fluctuating gate capacitance. Thus, the *SS* of the negative capacitance vacuum channel transistor could be approximately 20 mV/dec. In this paper, the negative capacitance vacuum channel transistor has been proven to be an important position in industrial applications.
